# Tear-film imaging measurements of the lipid and muco-aqueous layers and their association with dry eye disease signs and symptoms in adults: A pilot, prospective, cross-sectional study at a tertiary eye care center

**DOI:** 10.1371/journal.pone.0350397

**Published:** 2026-07-10

**Authors:** Isaac M. Tessone, Richard B. Rosen, Hernan Rios, Masako Chen, Alice Chandra Verticchio Vercellin, Brent A. Siesky, Paul A. Sidoti, Kira Manusis, Anna Fabczak-Kubicka, Keren Wood Shalem, Gal Antman

**Affiliations:** 1 Ophthalmology, New York Eye and Ear Infirmary of Mount Sinai, New York, New York, United States of America; 2 Ophthalmology, Icahn School of Medicine at Mount Sinai, New York, New York, United States of America; 3 Ophthalmology, Rutgers Robert Wood Johnson Medical School, New Brunswick, New Jersey, United States of America; 4 Ophthalmology, Rabin Medical Center, Petah Tikwa, Central, Israel; 5 Faculty of Medicine, Tel Aviv University, Tel Aviv, Israel; Universidad de Monterrey Division de Ciencias de la Salud, MEXICO

## Abstract

The purpose of this study was to assess tear film (TF) parameters in subjects with signs and symptoms of dry eye disease (DED) using a novel nanometer resolution tear film imager (TFI, AdOM, Israel). In this prospective, cross-sectional observational study, TF parameters, including muco-aqueous layer thickness (MALT) and lipid-layer thickness (LLT), were assessed in 134 eyes of 79 subjects. All participants underwent an anterior segment examination to assess for clinical signs of DED. Subjects completed the ocular surface disease index (OSDI) questionnaire to assess symptoms of DED. Following exclusions, the final analysis included 60 eyes from 34 subjects. A Mann-Whitney test was used for statistical comparisons, with p < 0.05 considered statistically significant. Participants with clinical signs of DED demonstrated significantly lower mean LLT values (42.1nm + / − 13.6) compared to eyes without clinical signs of DED (64.1nm + / − 25.2), p < 0.001. Participants with symptoms of mild, moderate or severe DED had significantly lower mean MALT values (2,858nm + / − 911) compared to eyes without symptoms of DED (3,699nm + / − 1,184), p < 0.05. Our analysis of the human TF indicated that eyes presenting with signs of DED were associated with a thinner LLT, while symptomatic eyes were associated with a thinner MALT.

## Introduction

Dry eye disease (DED) is a common ailment with an estimated prevalence of 6.8% in the United States, reaching as high as 50% among the adult population [1,2]. In DED, multifactorial models point to pathologic alterations of the tear film (TF) due to evaporative water loss, hyperosmolarity-induced tissue damage, and loss of epithelial and goblet cells. These changes to the TF provoke a vicious cycle of early TF breakup leading to increased hyperosmolarity and progressive pathologic changes.

DED is often classified as evaporative or aqueous deficient. The evaporative component of DED is attributed to meibomian gland dysfunction (MGD) and tear film lipid layer (TFLL) deficiencies [3]. The aqueous deficient component of DED is believed to be due to alterations in the TF muco-aqueous layer [3]. Current consensus suggests most DED cases exist on a continuum, consisting of elements of both etiologies within a single disease [4].

Diagnosis of DED relies on evaluating a multitude of clinical signs and symptoms including: a comprehensive medical history, anterior segment ocular examination with a slit lamp biomicroscope, Schirmer’s test, tear break-up time, TF osmolarity, fluorescein staining of the cornea, and questionnaires that assess symptoms such as the ocular surface disease index (OSDI) [5]. Because symptoms are subjective and signs vary, subtyping is difficult, and patients are often grouped simply as DED positive or negative. [6]. In addition, management options are highly varied and without clear clinical consensus. Optimizing treatment by DED subtype and etiology is therefore uncommon with patients frequently grouped into only DED positive or negative categories.

Several novel tear film imaging devices have been recently developed to quantify aspects of the TF. The Keratograph 5M (Oculus, Wetzlar, Germany) has been used to quantify the TFLL, while the TearScience LipiView II Interferometer (J&J, TearScience Inc, Morrisville, NC) and the Ocular Surface Analyzer (SBM System, Orbassano, Torino, Italy) have been used to investigate meibomian glands using infrared light [7–9]. The tear film imager (TFI), (AdOM, Israel) is a novel device developed to measure the muco-aqueous layer thickness (MALT), lipid layer thickness (LLT), and other dynamic TF parameters [10,11]. These various imaging technologies generate unique and highly specific biomarkers, each with different specificity, repeatability, and reproducibility. It is thus important to carefully interpret biomarkers from different imaging devices when making comparisons as they do not generate interchangeable values.

Improving diagnosis and treatment for DED requires the establishment of novel non-invasive reliable biomarkers to assist clinicians in categorizing DED subtypes. In this pilot study, we use a novel TFI system to assess differences in the sublayers and characteristics of the human TF in participants with signs and symptoms of DED compared to healthy controls. We further characterize clinical factors that were found to significantly impact TF parameters and discuss their potential importance for the management of DED. The primary endpoints were both decreased MALT and LLT, measured by TFI, in eyes with both signs or symptoms of dry eye disease to healthy controls.

## Materials and methods

This cross-sectional study was conducted between December 5, 2022 and June 8, 2023 at the New York Eye and Ear (NYEE) Infirmary of Mount Sinai, New York, NY, USA. All participants signed a written informed consent form. The study was conducted in accordance with the Declaration of Helsinki and the study protocol was approved by the Institutional Review Board of Icahn School of Medicine at Mount Sinai, New York, NY, USA.

Seventy-nine adults (134 eyes) who underwent anterior segment evaluation in an outpatient ophthalmology clinic (NYEE, New York, NY, USA) were included in an observational pilot study. Inclusion criteria were adults aged 18 or older including healthy controls and persons with signs and symptoms of DED. Exclusion criteria included ocular intervention, scan, or examination in the previous 2 hours; use of any eye ointments or drops in the previous 2 hours; use of contact lenses in the previous 7 days; intraocular or orbital surgeries in the previous 6 months; laser eye procedures in the past 1 month; history of refractive procedures; history of corneal ectasia.

All TF scans were taken by one of three device operators trained by the company. Both eyes of a study participant were imaged and included in the analyses if qualified. All subjects completed the OSDI questionnaire and underwent an anterior segment examination by a U.S. board-certified ophthalmologist after the TFI imaging session was completed. A combined thermometer and hygrometer (model H5075, Govee) was used to monitor temperature and humidity in the room of the imaging sessions.

**Fig 1:**
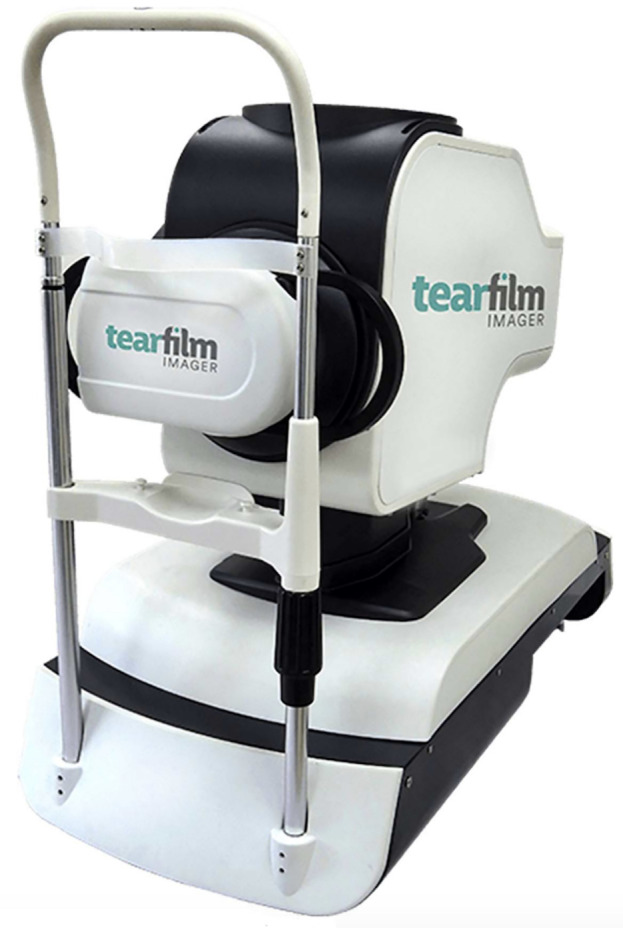
Tear film imager device.

The TFI device (Adom) used in our study employs spectral interference technology to image the pre-corneal surface with a large field of view (6.5 mm diameter) and high lateral resolution that measures static and dynamic parameters of the tear film during a single measurement [4,8] (Fig 1).

**Fig 2:**
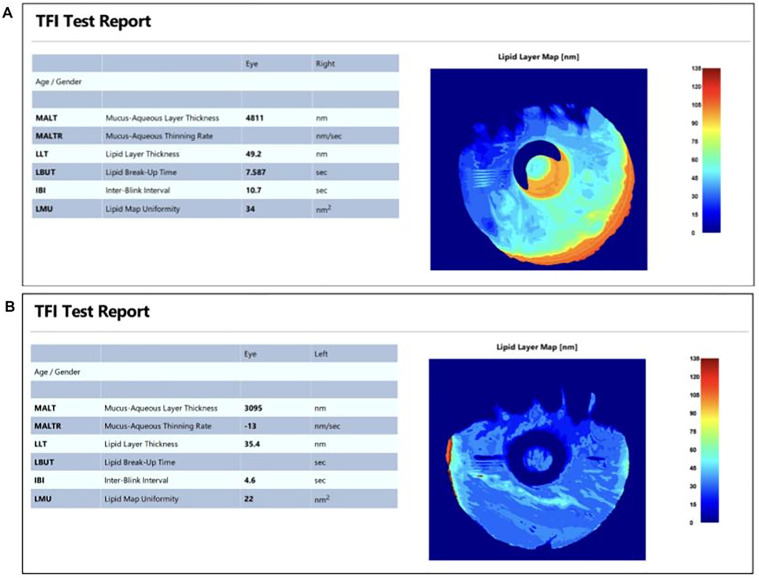
Tear Film Imager Report. A: Tear film Imager report showing the tear film parameters and a lipid layer heat map of the eye from a participant without any signs or symptoms of dry eyes disease (DED). B: Tear film Imager report showing the tear film parameters and a lipid layer heat map of an eye from a participant with signs and symptoms of DED). MALT: muco-aqueous layer thickness; MALTR: average slope of the MALT over time; LLT: lipid layer thickness; LBUT: lipid break-up time; IBI: inter-blink interval; LMU: lipid map uniformity.

The device produces a report of TF parameters based on a 40 second scan (**Fig 2).**

The parameters generated include: MALT, calculated as the average of the MALT values measured during the quasi- stable blink period (excluding instabilities before and after blinking) in nanometers; MALTR, the average slope of the MALT time trend in nanometers per second; LLT, the average of the LLT values measured during the quasi- stable blink period (excluding instabilities before and after blinking) in nanometers; IBI, the average time lapse between consecutive blinks; and LMU, variance of lipid layer thickness across five regions-of-interest of the lipid thickness map. As a result of blinks, fixation losses, and movements, the actual test duration of the scans are often shorter than 40 seconds. In instances where certain parameters were unable to be calculated, the analysis was conducted with available data. The device has previously demonstrated a consistent reproducibility with a Pearson coefficient of 0.88 for the MALT parameter [10].

Participants who reported the use of lotions, or make-up products around the eyes were retrospectively excluded from the analysis. This was decided after we observed during analysis that many participants with remarkably oily ocular surfaces (as recorded by the TFI device video) self-reported the use of lotion products on their face and around their eyes. Scans with unreliable measurements or a test duration below 6 seconds threshold were also excluded, as durations below this threshold were insufficient for complete TF analysis as per the device manufacturer, leaving 60 qualified eyes from 34 participants.

**Ethics statement:** The study protocol was approved by the Institutional Review Board committee at the Icahn School of Medicine at Mount Sinai, New York, NY. All patients signed an informed consent prior to initiation of this study, which adhered to the tenets of the Declaration of Helsinki.

### TF parameters and signs of DED

Qualified eyes were divided into two groups based on the presence or absence of clinical signs of DED. TF parameters were then compared between the two groups. For the purposes of this study, signs of DED were defined as evidence of MGD or anterior blepharitis on slit-lamp exam by a board-certified ophthalmologist. Signs of MGD consisted of plugging of gland orifices, altered meibum expression (absent, reduced, or hyperviscous), lid margin telangiectasias,and an irregular lid margin [12]. Signs of anterior blepharitis consisted of eyelash collarettes, scurf, and crusting of the eyelashes [13]. One of three board-certified ophthalmologists documented the presence of any signs of MGD or anterior blepharitis without masking. There were 27 eyes that demonstrated evidence of MGD, 29 without MGD, and 4 eyes which were undetermined for presence of MGD. The primary endpoints included decreased mucoaqueous layer thickness and lipid layer thickness in eyes with signs of DED.

### TF parameters and symptoms of DED

The same eyes were then divided into two groups based on symptoms of DED and TF parameters, compared for comparison. Quantification of symptoms of DED was based on the administered OSDI questionnaire, the most widely used survey tool for DED clinical trials [5]. The OSDI measures symptom frequency and triggers and vision-related quality of life. In accordance with the clinical interpretation of the OSDI score, we categorized a score ≤12 as a normal ocular surface, a score >12 and ≤22 as mild DED symptoms, a score >22 and ≤32 as moderate DED symptoms, and a score >32 as severe DED symptoms. To ensure sample comparability we included subjects with mild, moderate, and severe symptoms into one disease group (OSDI>12) yielding 50 eyes with an OSDI ≤12 and 10 eyes with an OSDI >12. The primary endpoints included decreased mucoaqueous layer thickness and lipid layer thickness in eyes with symptoms of DED.

### TF parameters comparison in males and females

The same cohorts of eyes were also divided into two groups based on biological sex. TF parameters were then compared between groups. This analysis was repeated with age-matching.

### Statistical analysis

For this pilot analysis, a Mann-Whitney test was used for statistical comparisons with p < 0.05 considered statistically significant. Each eye was treated as an independent observation and inter-eye correlation was not addressed, which may inflate significance. The small sample size was a limitation to pursuing further comprehensive statistical analyses such as sensitivity analyses and subgroup analyses. Moreover, given the exploratory nature of this pilot study, more advanced statistical approaches—such as multivariable models to adjust for age and temperature, repeatability analyses, and calculation of standardized effect sizes—were not performed. Consequently, results should be interpreted as preliminary and hypothesis-generating.

## Results

Overall baseline characteristics of the study population are shown in **Table 1**.

**Table 1:** Baseline Characteristics of Study Population. °C: Celsius degrees; DED Treatment: Dry eye disease treatment was defined by the use of artificial tears at least once per day or punctal plugs received in the past 6 months; MGD: Meibomian gland dysfunction on anterior segment examination; OSDI: Ocular Surface Disease Questionnaire Index (the cutoff of 12 was used as this represents the accepted threshold for normal vs symptomatic ocular surface pathology symptoms); TGM: Topical glaucoma medications. N: number.

Biological Sex N	Female	15 (25%)
	**Male**	45 (75%)
**Age (years)**	**Mean (SD)**	52 (19)
**MGD or Blepharitis N**	**No**	30 (50%)
	**Yes**	26 (43%)
	**Unknown**	4 (7%)
**OSDI Score**	**Mean (SD)**	7.5 (12.7)
	**≤ 12**	50 (83%)
	**> 12**	10 (17%)
**DED Treatment N**	**No**	51 (85%)
	**Yes**	9 (15%)
**Use of TGM N**	**No**	51 (84%)
	**Yes**	9 (16%)
**Room temperature (°C)**	**Mean (SD)**	22.4 (0.9)
**Humidity**	**Mean (SD)**	27.4 (5.7)

**Table 2** shows the TF parameters of eyes without signs of DED versus eyes with signs of DED. LLT in eyes with signs of DED (42.1nm (+/ − 13.6)) was significantly lower than in eyes without signs of MGD (64.1nm (+/ − 25.2)), p < 0.001. OSDI, age, room temperature, and LMU were also significantly different between the two groups (Table 2).

**Table 2:** Tear Film Imager parameters in eyes with DED signs compared to eyes without DED signs. °C: Celsius degrees; IBI: Inter-blink interval; LLT: Lipid layer thickness; LMU: Lipid map uniformity; MALT: Muco-aqueous layer thickness; MALTR: MALTR thinning rate; MGD: Meibomian gland dysfunction on anterior segment examination; OSDI: Ocular Surface Disease Questionnaire Index (the cutoff of 12 was used as this represents the accepted threshold for normal vs symptomatic ocular surface pathology symptoms; SD: Standard deviation (where applicable). *Statistically significant difference between the two groups.

Parameter	Eyes without DED signs (No MGD or blepharitis) (+/ − SD)	Eyes with DED signs (MGD or blepharitis) (+/ − SD)	p-value
N (eyes)	29	27	---
OSDI Score	4.2 (9.2)	11.5 (15.5)	0.02*
Age (years)	62.8 (15.1)	41.9 (17.1)	<0.001*
Temperature (°C)	22.0 (0.6)	23.0 (1.0)	<0.001*
Humidity (%)	27.8 (6.6)	27.1 (4.9)	0.690
MALT (nm)	3445 (1001)	3677 (1426)	0.780
MALTR (nm/sec)	−55 (53)	−66 (81)	0.974
LLT (nm)	64.7 (25.4)	42.2 (13.3)	<0.001*
LMU (nm^2^)	108 (195)	43 (105)	0.018*
IBI (sec)	5.7 (2.3)	7.5 (4.6)	0.461

**Table 3** shows the TF parameters of eyes without symptoms of DED versus eyes with symptoms of DED. Mean MALT in eyes with mild to severe symptoms was significantly lower (2,858 nm (+/ − 911)) than in eyes without symptoms (3,699 nm (+/ − 1,184)), p < 0.05 with a mean difference of 841nm.

**Table 3:** Tear film parameters in eyes with DED symptoms compared to eyes without DED symptoms. °C: Celsius; IBI: Inter-blink interval; LLT: Lipid layer thickness; LMU: Lipid map uniformity; MALT: Muco-aqueous layer thickness; MALTR: MALTR thinning rate; MGD: Meibomian gland dysfunction on anterior segment examination; OSDI: Ocular Surface Disease Questionnaire Index (the cutoff of 12 was used as this represents the accepted threshold for normal vs symptomatic ocular surface pathology symptoms; SD: Standard deviation (where applicable); *Statistically significant difference between the two groups.

Parameter	Eyes without symptoms of DED (OSDI≤ 12) (+/ − SD)	Eyes with symptoms of DED (OSDI>12) (+/ − SD)	p-value
N (eyes)	50	10	---
OSDI Score	2.8 (3.5)	31.0 (16.0)	<0.001*
Age (years)	50.7 (20.4)	57.6 (12.2)	0.546
Temperature (°C)	22.4 (0.9)	22.7 (0.8)	0.194
Humidity (%)	27.4 (6.2)	27.7 (3.2)	0.251
MALT (nm)	3699 (1184)	2858 (911)	0.039*
MALTR (nm/sec)	−62 (70)	−52 (58)	0.901
LLT (nm)	52.1 (22.8)	55.3 (25.3)	0.935
LMU (nm^2^)	62 (139)	125 (222)	0.479
IBI (sec)	6.7 (3.8)	5.9 (3.7)	0.301

**Table 4** shows the TF parameters in eyes from female participants compared to male participants. LLT is statistically significantly higher in females (65.8nm (+/ − 24.6)) compared to males (48.3nm (+/ − 21.0)), p = 0.004 with a mean difference of 17.5nm. The results also demonstrate statistically significant differences in age and temperature between the two groups.

**Table 4:** Tear film parameters in eyes from female participants compared to male participants. °C: Celsius; IBI: Inter-blink interval; LLT: Lipid layer thickness; LMU: Lipid map uniformity; MALT: Muco-aqueous layer thickness; MALTR: MALTR thinning rate; MGD: Meibomian gland dysfunction on anterior segment examination; OSDI: Ocular Surface Disease Questionnaire Index (the cutoff of 12 was used as this represents the accepted threshold for normal vs symptomatic ocular surface pathology symptoms; SD: Standard deviation (where applicable); *Statistically significant difference between the two groups.

Parameter	Male (+/ − SD)	Female (+/ − SD)	p-value
N (eyes)	45	15	---
OSDI Score	5.5 (9.6)	13.7 (18.3)	0.094
Age (years)	48.4 (21.0)	62.2 (7.0)	0.031*
Temperature (°C)	22.6 (0.9)	21.9 (0.6)	0.022*
Humidity (%)	27.2 (6.2)	28.1 (4.4)	0.361
MALT (nm)	3595 (1217)	3448 (1091)	0.778
MALTR (nm/sec)	−65 (69)	−45 (62)	0.286
LLT (nm)	48.3 (21.0)	65.8 (24.6)	0.004*
LMU (nm^2^)	49 (98)	146 (252)	0.242
IBI (sec)	6.7 (4.1)	6.2 (2.7)	0.772

Lastly, an age-matched analysis was conducted comparing 13 male eyes to 13 and female eyes and demonstrated no statistically significant differences in all TF parameters.

## Discussion

The diagnosis and medical management of DED is complicated by a heterogeneous set of clinical signs and symptoms that differ. Furthermore, it is difficult to pinpoint whether the underlying etiology of individual cases is evaporative or aqueous deficient, making DED subtyping frequently challenging. These challenges highlight the need for novel and quantitatively objective, non-invasive biomarkers. In this pilot study we used a novel TFI to assess the MALT and LLT in persons with signs and symptoms of DED.

We first analyzed the TF parameters in participants with signs of DED, as defined by the presence of MGD on clinical examination. Meibomian glands are believed to be responsible for lipid secretions to the TF, resulting in formation of the outer lipid layer of the TF, which helps prevent evaporation of the underlying aqueous layer. MGD has been demonstrated to be a leading cause of dry eye, specifically contributing to the evaporative component. Two major categories of MGD have been proposed: low-delivery MGD, which includes obstructive MGD, and high-delivery, hypersecretory MGD [14,15].

A thinner LLT has been previously reported in patients with MGD [16]. Using the LipiView™ Interferometer, Kim et al demonstrated significantly lower LLT values in MGD patients compared to controls, with a mean LLT of 52.25 nm and 88.51 nm, respectively [17]. They also demonstrated that a similar relationship existed between patients with DED compared to patients with a combination of both DED and MGD: patients with only DED had significantly higher LLT values compared to patients with both DED and MGD. In contrast, a study by Jung et al using a LipiView Interferometer demonstrated lower LLT values in controls compared to patients with DED with obstructive or hypersecretory MGD [18]. The authors suggested a hypothesis to explain this discrepancy was the significantly younger age of the controls compared to the DED patients. The authors confirmed their hypothesis by demonstrating that median LLT values for healthy controls was significantly higher compared to patients of the same age with obstructive MGD.

In our analysis, eyes from participants presenting with signs of DED were associated with significantly thinner LLTs than participants without signs of DED (**Table 2**), a finding consistent with previous studies [16,17]. This is understandable, as MGD compromises the glands’ ability to produce lipids, which are essential for maintaining the thickness and integrity of the tear film’s lipid layer.

There were statistically significant differences in age between the two groups, with the MGD group being significantly younger than the controls (42 years old vs. 63 years old, respectively, p **< 0.001**). This is noteworthy given that increasing age is thought to contribute to higher LLT values [18]. It can be argued that perhaps older age, and not lack of MGD, was contributory to increased LLT in our analysis. Furthermore, our analysis also showed that participants with MGD had significantly lower LMUs, indicating a more uniform lipid layer in this group. It is difficult to determine whether LLT, age, or the presence of MGD signs is most contributory to the decreased LMU. The clinical significance of this finding is unclear and further investigation with adjusted, multivariate models is warranted. Additionally, room temperature differed slightly between groups. However the mean difference was small at < 1° Celsius and therefore may not have a clinically meaningful impact on TF parameters. Previous studies have only shown TF changes with significantly larger temperature variations [19]. Future studies should adjust for temperature to exclude residual confounding.Considering symptoms of DED and TF parameters, our results highlighted significantly higher MALT values in participants with normal OSDI scores compared to mild, moderate, or severe OSDI scores (Table 3). We did not find significant differences in LLT or in the other TF parameters between the two groups (Table 3). Previous work on the minimal clinically important difference (MCID) for the OSDI has reported a range of 7.0 to 9.9 for all OSDI categories, with a MCID of 4.5 to 7.3 for mild or moderate disease and 7.3 to 13.4 for severe disease [20]. Our analysis showed mean OSDI of 2.8 and 31.0 in the groups without and with DED symptoms, respectively, suggestive of a clinically important difference in symptoms on average in the two groups analyzed in our study. A previous TFI study by Segev et al demonstrated significant differences in OSDI and MALT between controls and patients with DED, suggesting a potential relationship between OSDI and MALT [10]. They also demonstrated significant correlation between MALT and Schirmer’s Test. Patients with aqueous deficient DED had significantly higher OSDI scores and significantly lower Schirmer test values compared to controls, suggesting the presence of DED symptoms in eyes with an aqueous deficient component [17]. Taken together, these results suggest MALT may be the TF biomarker most associated with symptoms of DED. However, it is difficult to determine from this data whether the reduction in MALT is the cause of DED symptoms or if other factors are responsible for both the decrease in MALT and the presence of DED symptoms.

In our study, we also compared TF parameters between biological sexes. A previous study by Lee et al demonstrated higher proportions of thicker LLT in female patients using the LipiView Interferometer [21]. Jung et al demonstrated a positive association between female sex and LLT thickness and also between increasing age and LLT thickness. However, they suggest that a lower quality lipid layer with possible contamination in older female patients might have affected the LLT measurements [18]. Others have shown that tear film stability decreases with age [22,23]. Specifically, Maïssa and Guillon, using a Tearscope, demonstrated a significantly poorer quality of the LL and lower LLT values in women who were 45 years or older [24]. In our analysis, we initially observed higher LLT values in females compared to males, although the significantly higher age of the female group should be noted. Specifically, in an age-matched subgroup analysis we did not find any significant differences in TF parameters between male and female eyes. A limitation in this subgroup analysis is that the sample size was relatively small (n = 13 eyes per group). Our pilot study has several limitations.Firstly, participants with MGD being significantly younger than participants without MGD (Table 2). This poses the question of whether the thicker LLT is due to the lack of MGD or due to older age. An age-matched analysis was not possible due to the small sample size and minimal overlap in age between the groups. Future studies with larger sample sizes are needed to further the understanding of the relationship between MGD, age, and LLT. Secondly, approximately two-thirds of participant eyes were male. Given the role that biological sex is thought to play in the TF, this finding makes our results less generalizable to the general population. Thirdly, the grading of MGD is subjective and introduces a degree of variability and non-uniformity, although this applies for all current studies investigating MGD. Lastly, the small sample size and pilot nature of this study precluded more advanced statistical analyses, such as inter-eye correlation, subgroup, multivariate, repeatability analyses, and formal effect size calculations. As such, the significance of our findings may be overestimated and caution should be implemented when interpreting our results. Future confirmatory studies with larger sample sizes, repeatability and sensitivity analyses, and employment of mixed-effects models are needed to comprehensively characterize the relationship between MGD, age, and LLT.

Our pilot data highlights how several possible factors that potentially represent confounders should be considered when assessing the human TF, including age, sex, and the use of lotions and creams. Therefore, future research should include well-designed age- and sex-matched studies with larger sample sizes to further extrapolate our preliminary finding of TF changes in DED and identify and confirm utility of biomarkers for improving DED management.

In conclusion, our pilot study suggests that nanometer-resolution imaging of the TF using a non-invasive TFI may provide increased specificity for diagnosing DED subtype. Specifically, in participants with signs of DED, LLT was found to be significantly thinner than in participants without signs of DED, and in patients with symptoms of DED, MALT was found to be significantly thinner than in participants without symptoms of DED.
