# Supplementary material for: Tear-film imaging measurements of the lipid and muco-aqueous layers and their association with dry eye disease signs and symptoms in adults: A pilot, prospective, cross-sectional study at a tertiary eye care center
Source: PLoS One. 2026 Jul 10;21(7):e0350397. doi: 10.1371/journal.pone.0350397 (PMC13353963; doi:10.1371/journal.pone.0350397)
Supplement: Attachment — Submitted filename: Response to reviewers 04_28.docx [file pone.0350397.s003.docx]

**Response to Review:** “Assessing tear-film parameters in participants with signs and symptoms of dry eye disease using a novel tear-film imaging device”

**Manuscript No:** PONE-D-25-54074R1

**We would like to sincerely thank the editors and reviewers of PLOS One for your thoughtful and detailed reviews. We agree with all comments and suggestions provided and have revised the manuscript according to each reviewer’s concerns and addressed them below accordingly. We thank you for your consideration on behalf of all authors.**

Reviewer Comments:

The authors have responded thoughtfully to the reviewers’ suggestions and provided useful clarifications regarding the statistical limitations. The acknowledgment of the exploratory nature of the study is appreciated. Nevertheless, the choice to treat each eye as an independent observation without accounting for inter-eye correlation remains a methodological limitation that could affect the interpretation of the results. To ensure transparency and to help readers properly contextualize the findings, I recommend explicitly identifying the work as a pilot study in the title. For example: Tear-film imaging measurements of the lipid and muco-aqueous layers and their association with dry eye disease signs and symptoms in adults: A pilot, prospective, cross-sectional study at a tertiary eye care center. This adjustment would align the title with the authors’ own description of the study as preliminary and exploratory and would guide readers to interpret the results appropriately.

**Response: We appreciate this suggestion and agree that explicitly identifying the study as a pilot in the title would enhance transparency and help readers appropriately contextualize our findings. We have therefore revised the title and noted in the discussion the pilot nature of the investigation as recommended.**

The abstract reports results based on 134 eyes from 79 participants, but after exclusions the final analysis included 60 eyes from 34 subjects. This should be stated clearly in the abstract to avoid confusion and ensure consistency with the methods and results sections.

**Response: We agree that the abstract should clearly reflect the final analytic sample to maintain consistency across the main body’s methods and results sections. We have now revised the abstract to explicitly clarify this. We thank the reviewer for helping us improve the clarity of our manuscript.**
