# Supplementary material for: Tear-film imaging measurements of the lipid and muco-aqueous layers and their association with dry eye disease signs and symptoms in adults: A pilot, prospective, cross-sectional study at a tertiary eye care center
Source: PLoS One. 2026 Jul 10;21(7):e0350397. doi: 10.1371/journal.pone.0350397 (PMC13353963; doi:10.1371/journal.pone.0350397)
Supplement: Attachment — Submitted filename: Response to reviewers 04_01.docx [file pone.0350397.s002.docx]

**Response to Review:** “Assessing tear-film parameters in participants with signs and symptoms of dry eye disease using a novel tear-film imaging device”

**Manuscript No:** PONE-D-25-54074

**We would like to sincerely thank the editors and reviewers of PLOS One for your thoughtful and detailed reviews. We agree with the comments and suggestions provided and have revised the manuscript according to each reviewer’s concerns and addressed them below accordingly. We thank you for your consideration on behalf of all authors.**

**Journal Requirements:**

When submitting your revision, we need you to address these additional requirements.

1. Please ensure that your manuscript meets PLOS ONE's style requirements, including those for file naming. The PLOS ONE style templates can be found at

[https://journals.plos.org/plosone/s/file?id=wjVg/PLOSOne_formatting_sample_main_body.pdf](https://track.editorialmanager.com/CL0/https:%2F%2Fjournals.plos.org%2Fplosone%2Fs%2Ffile%3Fid=wjVg%2FPLOSOne_formatting_sample_main_body.pdf/1/010f019c5a1f1e07-21ec5284-d5e3-4c46-bd1d-90f341416240-000000/9OOgwu4DylCiEIYGNPk8kUxqCnSr9_bDWpzcrMlXwlE=250) and

[https://journals.plos.org/plosone/s/file?id=ba62/PLOSOne_formatting_sample_title_authors_affiliations.pdf](https://track.editorialmanager.com/CL0/https:%2F%2Fjournals.plos.org%2Fplosone%2Fs%2Ffile%3Fid=ba62%2FPLOSOne_formatting_sample_title_authors_affiliations.pdf/1/010f019c5a1f1e07-21ec5284-d5e3-4c46-bd1d-90f341416240-000000/cNiWVf_1E4_P1jP1al3fsb0GZghcsD9DCcRZEhMxdis=250)

**Response: Reviewed and correct formatting confirmed.**

2. Thank you for stating the following financial disclosure:

“NYEE Foundation grant”

Please state what role the funders took in the study.  If the funders had no role, please state: "The funders had no role in study design, data collection and analysis, decision to publish, or preparation of the manuscript."

If this statement is not correct you must amend it as needed.

Please include this amended Role of Funder statement in your cover letter; we will change the online submission form on your behalf.

**Response: Thank you for the guidance, corrections made (line 28)**

3. Please expand the acronym “NYEE” (as indicated in your financial disclosure) so that it states the name of your funders in full.

This information should be included in your cover letter; we will change the online submission form on your behalf.

**Response: Corrections made.**

4. In the online submission form you indicate that your data is not available for proprietary reasons and have provided a contact point for accessing this data. Please note that your current contact point is a co-author on this manuscript. According to our Data Policy, the contact point must not be an author on the manuscript and must be an institutional contact, ideally not an individual. Please revise your data statement to a non-author institutional point of contact, such as a data access or ethics committee, and send this to us via return email. Please also include contact information for the third party organization, and please include the full citation of where the data can be found.

**Response: Thank you for the guidance, corrections made.**

5. Your ethics statement should only appear in the Methods section of your manuscript. If your ethics statement is written in any section besides the Methods, please move it to the Methods section and delete it from any other section. Please ensure that your ethics statement is included in your manuscript, as the ethics statement entered into the online submission form will not be published alongside your manuscript.

**Response: Included and corrected, thank you for the feedback (line 153)**

6. If the reviewer comments include a recommendation to cite specific previously published works, please review and evaluate these publications to determine whether they are relevant and should be cited. There is no requirement to cite these works unless the editor has indicated otherwise.

**Response: Reviewed.**

**Additional Editor Comments:**

Your study addresses an important and clinically relevant topic in tear film imaging. I would encourage you to clarify two methodological aspects that would strengthen the transparency and statistical rigor of the manuscript.

First, please clarify how inter-eye correlation was handled in the statistical analysis. If both eyes from the same subjects were included, appropriate statistical methods that account for within-subject correlation should be applied. Alternatively, selecting a single eye per participant would avoid pseudo-replication. As currently written, the analysis appears to treat eyes as independent observations, which may overestimate statistical significance.

Second, please revise the abstract and methods to clearly state the final number of eyes and subjects included in the analysis, particularly if exclusions occurred due to external interferences or data quality concerns. The current wording suggests that 134 eyes from 79 subjects were analyzed, and any reduction in the effective sample should be transparently reported.

Addressing these two aspects will substantially improve the clarity, statistical validity, and interpretability of the study.

**Response: We appreciate the reviewer's thoughtful comments regarding statistical rigor and methodological transparency. We acknowledge that the inter-eye correlation was not formally addressed in our analysis. However, we respectfully note that this study was designed as a pilot and exploratory investigation to assess the feasibility of tear film imaging techniques and generate preliminary data for hypothesis generation. As such we felt it was beyond the scope of this analysis. Secondly, given our already limited sample size, restricting the analysis to one eye per participant would leave us with 34 eyes (instead of 60) in our final analysis. This would substantially reduce statistical power to the point where meaningful patterns and trends would be difficult to discern, thereby undermining the exploratory nature of this work.**

**To ensure full transparency, we made note in the methods section of the final number of eyes and subjects analyzed, including any exclusions due to data quality concerns (lines 149-151).**

**We also now clearly acknowledge in the methods section that eyes were treated as independent observations (lines 193-200). Additionally, in the limitations section of our discussion, we more appropriately contextualize our findings and elaborate on our statistical limitations and the potential direction of future studies (lines 324-330).**

5. Review Comments to the Author

Reviewer #1: In this different device study the authors researched the novel TFI system to assess differences in the sublayers and characteristics of the human TF in participants with signs and symptoms of DED compared to healthy controls. But I could not understand the device principles I think you should use some pictures of the devices. Also introduction is too long and different from the aim you should reorganize the section. Tables are too busy some results are give in the text.

**Response: We thank the reviewer for this suggestion and have now included a figure (Fig 1) of the device as recommended (line 126). We make note of the underlying principles of the imaging technique in the methods section (lines 120-126). However, we respectfully chose not to provide extensive technical detail regarding the device mechanisms, as this falls beyond the scope of our clinical pilot study. We now also include citations to prior publications that were more focused on the device's optical and mechanical principles for readers seeking more detailed technical information (References 4, 8, 10). We now also condensed and reorganized the introduction section to better align with our study aims, and only highlight the most noteworthy findings in our results to reduce redundancy between text and tabular data.**

Reviewer #2: 1. Causal language. The abstract's final sentence could be read as mechanistic inference ("suggests eyes,….. have a thinner LLT/MALT"). Keep strictly descriptive given the cross-sectional design. Rephrase to "were associated with" and move mechanistic speculation to Discussion.

**Response: Reviewed and corrected (lines 51-53)**

2. Ensure the OSDI validation paper is cited where OSDI is introduced and used (now only referenced generically in Introduction).Add recent work on TFI repeatability and LLT/MALT reproducibility if available, to contextualize measurement precision.

**Response: Reviewed and corrected (OSDI in line 76, line 175, reference #5, reproducibility in lines 135-138, reference #10)**

3. EQUATOR-aligned, STROBE-style title options that state the exposure/measure, outcome, population, setting, and design. Pick the one that best matches your manuscript. Eg: Tear Film Imager measurements of lipid and muco-aqueous layers and their association with dry eye signs and symptoms in adults: a prospective cross-sectional study at a tertiary eye clinic

**Response: Reviewed and corrected (lines 1-4)**

4. Use of both eyes without accounting for within-subject correlation. Both eyes were included whenever “qualified,” yet analyses use Mann–Whitney tests that assume independence. This risks underestimated variance and inflated significance. If reanalysis is infeasible, prespecify and use one eye per subject.

5. Post-hoc exclusions and risk of selection bias. Participants using lotions/makeup were “retrospectively excluded,” and scans shorter than 6 s were removed, leaving 60 eyes of 34 subjects. Criteria introduced after viewing data can bias results. Provide a CONSORT-style flow diagram, justify exclusions a priori, and present sensitivity analyses including the excluded eyes when possible. Define the 6-s threshold’s rationale and show results across alternative thresholds.

**Response: We appreciate the reviewer's thoughtful comments regarding statistical rigor. We acknowledge our methods may inflate significance. However, given our already limited sample size, further restricting our sample to one eye per participant would leave us with 34 eyes (instead of 60) in our final analysis which would substantially reduce statistical power to the point where meaningful patterns and trends would be difficult to discern, thereby undermining the exploratory nature of this work. We now make note of and elaborate on this statistical limitation (lines 193-200) and we more appropriately contextualize our findings and limitations (lines 323-330. The 6s rationale is per the device manufacturer. We now further clarify the threshold (lines 150-151). Additionally, we didn’t pursue sensitivity analyses and results with alternative thresholds as we felt this was beyond the scope of our pilot study (line 193-200).**

6. Confounding by age and environment not controlled. In page 16, Table 2 shows large age difference between groups (62.8 vs 41.9 years) and different room temperatures; page 20, the “DED signs” group is substantially younger, yet age is associated with LLT in prior literature and in your discussion. Temperature also differed. Adjust comparisons for age and temperature using multivariable models, or perform age-matched subgroup analyses with adequate power. Report standardized mean differences to quantify group imbalance.

**Response: We appreciate the reviewer's insights and guidance. We attempted the age-matching but there were no statistical differences found (possibly due to the low power). We adjusted our text to make note of this in the results (lines 230-231) and discussion (lines 311-312). We also made adjustments to elaborate on the relationship between temperature and TF parameters (lines 276-279). Regarding other statistical analyses, we deemed multivariable models beyond the scope of this given the limited sample size and exploratory nature of our analysis.**

7. Definition and grading of “DED signs” require clarity. “Signs of DED” are defined as MGD or anterior blepharitis, yet Table 2 labels the group “MGD”. The grading scale, inter-grader reliability, and masking are not reported. Specify diagnostic criteria and grades for MGD and blepharitis, whether a single masked examiner graded all cases, and provide inter-rater or test-retest reliability if applicable.

**Response: Reviewed and corrected (lines 163-170).**

8. Repeatability and measurement quality metrics are missing. No within-session or between-session repeatability (e.g., ICC, CoV) is provided for MALT, MALTR, or LLT. Without reliability, effect sizes are difficult to interpret. Add a repeatability analysis from duplicate measures or cite device reliability in a comparable setting, and state any quality-control rules used (e.g., blink detection, ROI rejection, minimum frames).

**Response: Thank you for this feedback. We now included literature on reproducibility on the device used in our study (lines 135-138, reference #10).**

9. Primary outcome and multiplicity not prespecified, there were multiple endpoints tested with Mann–Whitney. The study tests many TF parameters and subgroupings without a declared primary endpoint or multiple-testing control. Prespecify a single primary endpoint (for example, LLT difference between MGD vs no MGD), define secondary endpoints, and apply Holm or Benjamini–Hochberg adjustments. Report adjusted p values and 95% CIs.

10. Tables 2–4: Results rely on means, SDs, and p values only. Clinical interpretation would benefit from effect sizes. Add mean differences with 95% CIs, and where non-normal, provide Hodges–Lehmann median differences. Include standardized effect sizes.

**Response: We thank the reviewer for this important methodological comment and have now clearly specified our primary endpoints in the revised manuscript (lines 99-100, 169-170, 181-183). We also clarify mean differences (lines 223, 227). However, given the hypothesis-generating goals of this pilot study, we respectfully chose not to implement formal multiple-testing adjustments, effect size calculations, confidence intervals, or Hodges–Lehmann median differences. We felt such corrections would be more appropriate for confirmatory studies with predefined hypotheses, whereas our aim was to identify preliminary trends. We have acknowledged this limitation in the discussion and encourage readers to interpret our findings with caution (lines 323-330)**

11. The data availability statement mentioned on the opening pages and the manuscript contradict, please check.
12. Study design description in p.11, lines 49–52: The design is described as “prospective cross-sectional,” which can confuse readers. Suggested fix: State “prospective, cross-sectional observational study.”

**Response: Reviewed and corrected**

13. Sample size rationale (Methods overall). No precision or feasibility justification is provided. The authors need to add a brief rationale for the planned sample size or acknowledge as a limitation.

**Response: We thank the reviewer for this comment. As this was an exploratory pilot study, a formal sample size calculation was not performed. We have now acknowledged this as a limitation in the revised manuscript (lines 323-330).**

14. Use “muco-aqueous” consistently and define abbreviations once. Standardize the use of MALT and MALTR. MALTR has not been described in the paper. Figures have a heatmap. Does the heatmap signify MALT, MALTR, or TLL? Please mention in the figure notes. Figure 1A shows that there is no MALTR, and how missing data was accounted for

**Response: We included information on MALTR (line 129). The heatmap represents the LLT, which we now clarified (lines 139-142). In instances in which there was missing data (which was uncommon), we proceeded with the calculation as is. We now acknowledge this in the manuscript (line 135).**

15. Page 11: “eyes presenting with signs of DED have a thinner LLT, whereas eyes with symptoms of DED have a thinner MALT.” Suggested change “Eyes with clinical signs of DED showed thinner LLT, while symptomatic eyes showed thinner MALT.”
16. pg.12, “Due to the subjective nature… patients frequently grouped into only DED positive or negative categories.” Suggested change “Because symptoms are subjective and signs vary, subtyping is difficult, and patients are often grouped simply as DED positive or negative.”

**Response: Reviewed and adjusted (lines 51-52, lines 76-77)**

17. Page19: “The significance of this finding is unclear and further investigation is warranted.” Revision: “The clinical significance is unclear; please explore this in adjusted models or acknowledge as hypothesis-generating.”
18. Page 21: “We also found statistically significant differences in the room temperature…unlikely to significantly affect the results.” Change to: “Room temperature differed slightly between groups. Although the absolute difference was small, please adjust for temperature to exclude residual confounding.”

**Response: Reviewed and adjusted (line 274, lines 276-279)**

19. References that would improve the discussion: A recent overview of environmental influences and chamber conditions on tear metrics to contextualize temperature/humidity effects.

**Response: Reviewed and adjusted (lines 277-279, reference #19)**

20. A statistical reference for analyzing paired-eye data in ophthalmic studies. If available, more recent TFI reproducibility or validation studies in symptomatic DED cohorts.

**Response: Thank you for this insight. We adjusted the manuscript to reflect the reproducibility of the device used in the study(lines 136-138, reference #10). However in the interest of adhering to our pilot goals, we elected to limit analysis of paired-eye data in other ophthalmic studies.**
