# Supplementary material for: Tear-film imaging measurements of the lipid and muco-aqueous layers and their association with dry eye disease signs and symptoms in adults: A pilot, prospective, cross-sectional study at a tertiary eye care center
Source: PLoS One. 2026 Jul 10;21(7):e0350397. doi: 10.1371/journal.pone.0350397 (PMC13353963; doi:10.1371/journal.pone.0350397)
Supplement: Peer review history

# Peer review: Decision Letter 0

**DOI:** 10.1371/journal.pone.0350397.r001

**Authors:** Manuel Garza León

13 Feb 2026

PONE-D-25-54074

Assessing tear-film parameters in participants with signs and symptoms of dry eye disease using a novel tear-film imaging device

PLOS One

Dear Dr. Antman,

Thank you for submitting your manuscript to PLOS ONE. After careful consideration, we feel that it has merit but does not fully meet PLOS ONE’s publication criteria as it currently stands. Therefore, we invite you to submit a revised version of the manuscript that addresses the points raised during the review process.

Please submit your revised manuscript by Mar 30 2026 11:59PM. If you will need more time than this to complete your revisions, please reply to this message or contact the journal office at plosone@plos.org. When you're ready to submit your revision, log on to https://www.editorialmanager.com/pone/ and select the 'Submissions Needing Revision' folder to locate your manuscript file.

If you would like to make changes to your financial disclosure, please include your updated statement in your cover letter. Guidelines for resubmitting your figure files are available below the reviewer comments at the end of this letter.

If applicable, we recommend that you deposit your laboratory protocols in protocols.io to enhance the reproducibility of your results. Protocols.io assigns your protocol its own identifier (DOI) so that it can be cited independently in the future. For instructions see: https://journals.plos.org/plosone/s/submission-guidelines#loc-laboratory-protocols. Additionally, PLOS ONE offers an option for publishing peer-reviewed Lab Protocol articles, which describe protocols hosted on protocols.io. Read more information on sharing protocols at https://plos.org/protocols?utm_medium=editorial-email&utm_source=authorletters&utm_campaign=protocols.

We look forward to receiving your revised manuscript.

Kind regards,

Manuel Garza León

Academic Editor

PLOS One

**Journal Requirements:**

When submitting your revision, we need you to address these additional requirements.

1. Please ensure that your manuscript meets PLOS ONE's style requirements, including those for file naming. The PLOS ONE style templates can be found at

https://journals.plos.org/plosone/s/file?id=wjVg/PLOSOne_formatting_sample_main_body.pdf and

https://journals.plos.org/plosone/s/file?id=ba62/PLOSOne_formatting_sample_title_authors_affiliations.pdf

2. Thank you for stating the following financial disclosure:

“NYEE Foundation grant”

Please state what role the funders took in the study.  If the funders had no role, please state: "The funders had no role in study design, data collection and analysis, decision to publish, or preparation of the manuscript."

If this statement is not correct you must amend it as needed.

Please include this amended Role of Funder statement in your cover letter; we will change the online submission form on your behalf.

3. Please expand the acronym “NYEE” (as indicated in your financial disclosure) so that it states the name of your funders in full.

This information should be included in your cover letter; we will change the online submission form on your behalf.

4. In the online submission form you indicate that your data is not available for proprietary reasons and have provided a contact point for accessing this data. Please note that your current contact point is a co-author on this manuscript. According to our Data Policy, the contact point must not be an author on the manuscript and must be an institutional contact, ideally not an individual. Please revise your data statement to a non-author institutional point of contact, such as a data access or ethics committee, and send this to us via return email. Please also include contact information for the third party organization, and please include the full citation of where the data can be found.

5. Your ethics statement should only appear in the Methods section of your manuscript. If your ethics statement is written in any section besides the Methods, please move it to the Methods section and delete it from any other section. Please ensure that your ethics statement is included in your manuscript, as the ethics statement entered into the online submission form will not be published alongside your manuscript.

6. If the reviewer comments include a recommendation to cite specific previously published works, please review and evaluate these publications to determine whether they are relevant and should be cited. There is no requirement to cite these works unless the editor has indicated otherwise.

**Additional Editor Comments:**

Your study addresses an important and clinically relevant topic in tear film imaging. I would encourage you to clarify two methodological aspects that would strengthen the transparency and statistical rigor of the manuscript.

First, please clarify how inter-eye correlation was handled in the statistical analysis. If both eyes from the same subjects were included, appropriate statistical methods that account for within-subject correlation should be applied. Alternatively, selecting a single eye per participant would avoid pseudo-replication. As currently written, the analysis appears to treat eyes as independent observations, which may overestimate statistical significance.

Second, please revise the abstract and methods to clearly state the final number of eyes and subjects included in the analysis, particularly if exclusions occurred due to external interferences or data quality concerns. The current wording suggests that 134 eyes from 79 subjects were analyzed, and any reduction in the effective sample should be transparently reported.

Addressing these two aspects will substantially improve the clarity, statistical validity, and interpretability of the study.

[Note: HTML markup is below. Please do not edit.]

Reviewers' comments:

Reviewer's Responses to Questions

**Comments to the Author**

1. Is the manuscript technically sound, and do the data support the conclusions?

The manuscript must describe a technically sound piece of scientific research with data that supports the conclusions. Experiments must have been conducted rigorously, with appropriate controls, replication, and sample sizes. The conclusions must be drawn appropriately based on the data presented.

Reviewer #1: Yes

Reviewer #2: Yes

**********

2. Has the statistical analysis been performed appropriately and rigorously?

Reviewer #1: Yes

Reviewer #2: No

**********

3. Have the authors made all data underlying the findings in their manuscript fully available?

The PLOS Data policy requires authors to make all data underlying the findings described in their manuscript fully available without restriction, with rare exception (please refer to the Data Availability Statement in the manuscript PDF file). The data should be provided as part of the manuscript or its supporting information, or deposited to a public repository. For example, in addition to summary statistics, the data points behind means, medians and variance measures should be available. If there are restrictions on publicly sharing data—e.g. participant privacy or use of data from a third party—those must be specified.

Reviewer #1: Yes

Reviewer #2: No

**********

4. Is the manuscript presented in an intelligible fashion and written in standard English?

PLOS ONE does not copyedit accepted manuscripts, so the language in submitted articles must be clear, correct, and unambiguous. Any typographical or grammatical errors should be corrected at revision, so please note any specific errors here.

Reviewer #1: Yes

Reviewer #2: Yes

**********

5. Review Comments to the Author

Please use the space provided to explain your answers to the questions above. You may also include additional comments for the author, including concerns about dual publication, research ethics, or publication ethics. (Please upload your review as an attachment if it exceeds 20,000 characters)

Reviewer #1: In this different device study the authors researched the novel TFI system to assess differences in the sublayers and characteristics of the human TF in participants with signs and symptoms of DED compared to healthy controls. But I could not understand the device principles I think you should use some pictures of the devices. Also introduction is too long and different from the aim you should reorganize the section. Tables are too busy some results are give in the text.

Reviewer #2: 1. Causal language. The abstract's final sentence could be read as mechanistic inference ("suggests eyes,….. have a thinner LLT/MALT"). Keep strictly descriptive given the cross-sectional design. Rephrase to "were associated with" and move mechanistic speculation to Discussion.

2. Ensure the OSDI validation paper is cited where OSDI is introduced and used (now only referenced generically in Introduction).Add recent work on TFI repeatability and LLT/MALT reproducibility if available, to contextualize measurement precision.

3. EQUATOR-aligned, STROBE-style title options that state the exposure/measure, outcome, population, setting, and design. Pick the one that best matches your manuscript. Eg: Tear Film Imager measurements of lipid and muco-aqueous layers and their association with dry eye signs and symptoms in adults: a prospective cross-sectional study at a tertiary eye clinic

4. Use of both eyes without accounting for within-subject correlation. Both eyes were included whenever “qualified,” yet analyses use Mann–Whitney tests that assume independence. This risks underestimated variance and inflated significance. If reanalysis is infeasible, prespecify and use one eye per subject.

5. Post-hoc exclusions and risk of selection bias. Participants using lotions/makeup were “retrospectively excluded,” and scans shorter than 6 s were removed, leaving 60 eyes of 34 subjects. Criteria introduced after viewing data can bias results. Provide a CONSORT-style flow diagram, justify exclusions a priori, and present sensitivity analyses including the excluded eyes when possible. Define the 6-s threshold’s rationale and show results across alternative thresholds.

6. Confounding by age and environment not controlled. In page 16, Table 2 shows large age difference between groups (62.8 vs 41.9 years) and different room temperatures; page 20, the “DED signs” group is substantially younger, yet age is associated with LLT in prior literature and in your discussion. Temperature also differed. Adjust comparisons for age and temperature using multivariable models, or perform age-matched subgroup analyses with adequate power. Report standardized mean differences to quantify group imbalance.

7. Definition and grading of “DED signs” require clarity. “Signs of DED” are defined as MGD or anterior blepharitis, yet Table 2 labels the group “MGD”. The grading scale, inter-grader reliability, and masking are not reported. Specify diagnostic criteria and grades for MGD and blepharitis, whether a single masked examiner graded all cases, and provide inter-rater or test-retest reliability if applicable.

8. Repeatability and measurement quality metrics are missing. No within-session or between-session repeatability (e.g., ICC, CoV) is provided for MALT, MALTR, or LLT. Without reliability, effect sizes are difficult to interpret. Add a repeatability analysis from duplicate measures or cite device reliability in a comparable setting, and state any quality-control rules used (e.g., blink detection, ROI rejection, minimum frames).

9. Primary outcome and multiplicity not prespecified, there were multiple endpoints tested with Mann–Whitney. The study tests many TF parameters and subgroupings without a declared primary endpoint or multiple-testing control. Prespecify a single primary endpoint (for example, LLT difference between MGD vs no MGD), define secondary endpoints, and apply Holm or Benjamini–Hochberg adjustments. Report adjusted p values and 95% CIs.

10. Tables 2–4: Results rely on means, SDs, and p values only. Clinical interpretation would benefit from effect sizes. Add mean differences with 95% CIs, and where non-normal, provide Hodges–Lehmann median differences. Include standardized effect sizes.

11. The data availability statement mentioned on the opening pages and the manuscript contradict, please check.

12. Study design description in p.11, lines 49–52: The design is described as “prospective cross-sectional,” which can confuse readers. Suggested fix: State “prospective, cross-sectional observational study.”

13. Sample size rationale (Methods overall). No precision or feasibility justification is provided. The authors need to add a brief rationale for the planned sample size or acknowledge as a limitation.

14. Use “muco-aqueous” consistently and define abbreviations once. Standardize the use of MALT and MALTR. MALTR has not been described in the paper. Figures have a heatmap. Does the heatmap signify MALT, MALTR, or TLL? Please mention in the figure notes. Figure 1A shows that there is no MALTR, and how missing data was accounted for

15. Page 11: “eyes presenting with signs of DED have a thinner LLT, whereas eyes with symptoms of DED have a thinner MALT.” Suggested change “Eyes with clinical signs of DED showed thinner LLT, while symptomatic eyes showed thinner MALT.”

16. pg.12, “Due to the subjective nature… patients frequently grouped into only DED positive or negative categories.” Suggested change “Because symptoms are subjective and signs vary, subtyping is difficult, and patients are often grouped simply as DED positive or negative.”

17. Page19: “The significance of this finding is unclear and further investigation is warranted.” Revision: “The clinical significance is unclear; please explore this in adjusted models or acknowledge as hypothesis-generating.”

18. Page 21: “We also found statistically significant differences in the room temperature…unlikely to significantly affect the results.” Change to: “Room temperature differed slightly between groups. Although the absolute difference was small, please adjust for temperature to exclude residual confounding.”

19. References that would improve the discussion: A recent overview of environmental influences and chamber conditions on tear metrics to contextualize temperature/humidity effects.

20. A statistical reference for analyzing paired-eye data in ophthalmic studies. If available, more recent TFI reproducibility or validation studies in symptomatic DED cohorts.

**********

6. PLOS authors have the option to publish the peer review history of their article (what does this mean?). If published, this will include your full peer review and any attached files.

If you choose “no”, your identity will remain anonymous but your review may still be made public.

**Do you want your identity to be public for this peer review?**  For information about this choice, including consent withdrawal, please see our Privacy Policy.

Reviewer #1: No

Reviewer #2: No

**********

[NOTE: If reviewer comments were submitted as an attachment file, they will be attached to this email and accessible via the submission site. Please log into your account, locate the manuscript record, and check for the action link "View Attachments". If this link does not appear, there are no attachment files.]

To ensure your figures meet our technical requirements, please review our figure guidelines: https://journals.plos.org/plosone/s/figures

You may also use PLOS’s free figure tool, NAAS, to help you prepare publication quality figures: https://journals.plos.org/plosone/s/figures#loc-tools-for-figure-preparation.

NAAS will assess whether your figures meet our technical requirements by comparing each figure against our figure specifications.

# Author comment: Author response to Decision Letter 1

**DOI:** 10.1371/journal.pone.0350397.r002

4 Apr 2026

We would like to sincerely thank the editors and reviewers of PLOS One for your thoughtful and detailed reviews. We agree with the comments and suggestions provided and have revised the manuscript according to each reviewer’s concerns and addressed them below accordingly. We thank you for your consideration on behalf of all authors.

We attached a seperate document with the responses.

Attachment Submitted filename: Response to reviewers 04_01.docx

# Editor report: Decision Letter 1

**DOI:** 10.1371/journal.pone.0350397.r003

**Authors:** Manuel Garza León, Manuel Garza León

20 Apr 2026

PONE-D-25-54074R1

Assessing tear-film parameters in participants with signs and symptoms of dry eye disease using a novel tear-film imaging device

PLOS One

Dear Dr. Antman,

Thank you for submitting your manuscript to PLOS ONE. After careful consideration, we feel that it has merit but does not fully meet PLOS ONE’s publication criteria as it currently stands. Therefore, we invite you to submit a revised version of the manuscript that addresses the points raised during the review process.

Please submit your revised manuscript by Jun 04 2026 11:59PM. If you will need more time than this to complete your revisions, please reply to this message or contact the journal office at plosone@plos.org. When you're ready to submit your revision, log on to https://www.editorialmanager.com/pone/ and select the 'Submissions Needing Revision' folder to locate your manuscript file.

Please include the following items when submitting your revised manuscript:

- A letter that responds to each point raised by the academic editor and reviewer(s). You should upload this letter as a separate file labeled 'Response to Reviewers'.
- A marked-up copy of your manuscript that highlights changes made to the original version. You should upload this as a separate file labeled 'Revised Manuscript with Track Changes'.
- An unmarked version of your revised paper without tracked changes. You should upload this as a separate file labeled 'Manuscript'

If you would like to make changes to your financial disclosure, please include your updated statement in your cover letter. Guidelines for resubmitting your figure files are available below the reviewer comments at the end of this letter.

If applicable, we recommend that you deposit your laboratory protocols in protocols.io to enhance the reproducibility of your results. Protocols.io assigns your protocol its own identifier (DOI) so that it can be cited independently in the future. For instructions see: https://journals.plos.org/plosone/s/submission-guidelines#loc-laboratory-protocols. Additionally, PLOS ONE offers an option for publishing peer-reviewed Lab Protocol articles, which describe protocols hosted on protocols.io. Read more information on sharing protocols at https://plos.org/protocols?utm_medium=editorial-email&utm_source=authorletters&utm_campaign=protocols.

As the corresponding author, your ORCID iD is verified in the submission system and will appear in the published article. PLOS supports the use of ORCID, and we encourage all coauthors to register for an ORCID iD and use it as well. Please encourage your coauthors to verify their ORCID iD within the submission system before final acceptance, as unverified ORCID iDs will not appear in the published article. *Only*  the individual author can complete the verification step; PLOS staff *cannot*  verify ORCID iDs on behalf of authors.

We look forward to receiving your revised manuscript.

Kind regards,

Manuel Garza León

Academic Editor

PLOS One

Journal Requirements:

1. If the reviewer comments include a recommendation to cite specific previously published works, please review and evaluate these publications to determine whether they are relevant and should be cited. There is no requirement to cite these works unless the editor has indicated otherwise.

2. Please review your reference list to ensure that it is complete and correct. If you have cited papers that have been retracted, please include the rationale for doing so in the manuscript text, or remove these references and replace them with relevant current references. Any changes to the reference list should be mentioned in the rebuttal letter that accompanies your revised manuscript. If you need to cite a retracted article, indicate the article’s retracted status in the References list and also include a citation and full reference for the retraction notice.

Additional Editor Comments:

The authors have responded thoughtfully to the reviewers’ suggestions and provided useful clarifications regarding the statistical limitations. The acknowledgment of the exploratory nature of the study is appreciated. Nevertheless, the choice to treat each eye as an independent observation without accounting for inter-eye correlation remains a methodological limitation that could affect the interpretation of the results. To ensure transparency and to help readers properly contextualize the findings, I recommend explicitly identifying the work as a pilot study in the title. For example: Tear-film imaging measurements of the lipid and muco-aqueous layers and their association with dry eye disease signs and symptoms in adults: A pilot, prospective, cross-sectional study at a tertiary eye care center. This adjustment would align the title with the authors’ own description of the study as preliminary and exploratory, and would guide readers to interpret the results appropriately.

The abstract reports results based on 134 eyes from 79 participants, but after exclusions the final analysis included 60 eyes from 34 subjects. This should be stated clearly in the abstract to avoid confusion and ensure consistency with the methods and results sections.

[Note: HTML markup is below. Please do not edit.]

[NOTE: If reviewer comments were submitted as an attachment file, they will be attached to this email and accessible via the submission site. Please log into your account, locate the manuscript record, and check for the action link "View Attachments". If this link does not appear, there are no attachment files.]

To ensure your figures meet our technical requirements, please review our figure guidelines: https://journals.plos.org/plosone/s/figures

You may also use PLOS’s free figure tool, NAAS, to help you prepare publication quality figures: https://journals.plos.org/plosone/s/figures#loc-tools-for-figure-preparation.

NAAS will assess whether your figures meet our technical requirements by comparing each figure against our figure specifications.

# Author comment: Author response to Decision Letter 2

**DOI:** 10.1371/journal.pone.0350397.r004

29 Apr 2026

We would like to sincerely thank the editors and reviewers of PLOS One for your thoughtful and detailed reviews. We agree with all comments and suggestions provided and have revised the manuscript according to each reviewer’s concerns and addressed them below accordingly. We thank you for your consideration on behalf of all authors.

Reviewer Comments:

The authors have responded thoughtfully to the reviewers’ suggestions and provided useful clarifications regarding the statistical limitations. The acknowledgment of the exploratory nature of the study is appreciated. Nevertheless, the choice to treat each eye as an independent observation without accounting for inter-eye correlation remains a methodological limitation that could affect the interpretation of the results. To ensure transparency and to help readers properly contextualize the findings, I recommend explicitly identifying the work as a pilot study in the title. For example: Tear-film imaging measurements of the lipid and muco-aqueous layers and their association with dry eye disease signs and symptoms in adults: A pilot, prospective, cross-sectional study at a tertiary eye care center. This adjustment would align the title with the authors’ own description of the study as preliminary and exploratory and would guide readers to interpret the results appropriately.

Response: We appreciate this suggestion and agree that explicitly identifying the study as a pilot in the title would enhance transparency and help readers appropriately contextualize our findings. We have therefore revised the title and noted in the discussion the pilot nature of the investigation as recommended.

The abstract reports results based on 134 eyes from 79 participants, but after exclusions the final analysis included 60 eyes from 34 subjects. This should be stated clearly in the abstract to avoid confusion and ensure consistency with the methods and results sections.

Response: We agree that the abstract should clearly reflect the final analytic sample to maintain consistency across the main body’s methods and results sections. We have now revised the abstract to explicitly clarify this. We thank the reviewer for helping us improve the clarity of our manuscript.

Attachment Submitted filename: Response to reviewers 04_28.docx

# Editor report: Decision Letter 2

**DOI:** 10.1371/journal.pone.0350397.r005

**Authors:** Manuel Garza León, Manuel Garza León, Manuel Garza León

14 May 2026

Tear-film imaging measurements of the lipid and muco-aqueous layers and their association with dry eye disease signs and symptoms in adults: A pilot, prospective, cross-sectional study at a tertiary eye care center

PONE-D-25-54074R2

Dear Dr. Antman,

We’re pleased to inform you that your manuscript has been judged scientifically suitable for publication and will be formally accepted for publication once it meets all outstanding technical requirements.

Within one week, you’ll receive an e-mail detailing the required amendments. When these have been addressed, you’ll receive a formal acceptance letter and your manuscript will be scheduled for publication.

An invoice will be generated when your article is formally accepted. Please note, if your institution has a publishing partnership with PLOS and your article meets the relevant criteria, all or part of your publication costs will be covered. Please make sure your user information is up-to-date by logging into Editorial Manager at Editorial Manager® and clicking the ‘Update My Information' link at the top of the page. For questions related to billing, please contact billing support.

If your institution or institutions have a press office, please notify them about your upcoming paper to help maximize its impact. If they’ll be preparing press materials, please inform our press team as soon as possible -- no later than 48 hours after receiving the formal acceptance. Your manuscript will remain under strict press embargo until 2 pm Eastern Time on the date of publication. For more information, please contact onepress@plos.org.

Kind regards,

Manuel Garza León

Academic Editor

PLOS One

Additional Editor Comments (optional):

Reviewers' comments:

# Editor report: Acceptance letter

**DOI:** 10.1371/journal.pone.0350397.r006

**Authors:** Manuel Garza León, Manuel Garza León, Manuel Garza León

PONE-D-25-54074R2

PLOS One

Dear Dr. Antman,

I'm pleased to inform you that your manuscript has been deemed suitable for publication in PLOS One. Congratulations! Your manuscript is now being handed over to our production team.

At this stage, our production department will prepare your paper for publication. This includes ensuring the following:

* All references, tables, and figures are properly cited

* All relevant supporting information is included in the manuscript submission,

* There are no issues that prevent the paper from being properly typeset

You will receive further instructions from the production team, including instructions on how to review your proof when it is ready. Please keep in mind that we are working through a large volume of accepted articles, so please give us a few days to review your paper and let you know the next and final steps.

Lastly, if your institution or institutions have a press office, please let them know about your upcoming paper now to help maximize its impact. If they'll be preparing press materials, please inform our press team within the next 48 hours. Your manuscript will remain under strict press embargo until 2 pm Eastern Time on the date of publication. For more information, please contact onepress@plos.org.

You will receive an invoice from PLOS for your publication fee after your manuscript has reached the completed accept phase. If you receive an email requesting payment before acceptance or for any other service, this may be a phishing scheme. Learn how to identify phishing emails and protect your accounts at https://explore.plos.org/phishing.

If we can help with anything else, please email us at customercare@plos.org.

Thank you for submitting your work to PLOS ONE and supporting open access.

Kind regards,

PLOS ONE Editorial Office Staff

on behalf of

Dr. Manuel Garza León

Academic Editor

PLOS One
